# Editorial: O-GlcNAcylation and the immune system

**DOI:** 10.3389/fimmu.2024.1474042

**Published:** 2024-08-20

**Authors:** Gerald W. Hart, Parameswaran Ramakrishnan

**Affiliations:** ^1^ Complex Carbohydrate Research Center, University of Georgia, Athens, GA, United States; ^2^ Department of Biochemistry and Molecular Biology, University of Georgia, Athens, GA, United States; ^3^ Department of Pathology, School of Medicine, Case Western Reserve University, Cleveland, OH, United States; ^4^ The Case Comprehensive Cancer Center, School of Medicine, Case Western Reserve University, Cleveland, OH, United States; ^5^ Department of Biochemistry, School of Medicine, Case Western Reserve University, Cleveland, OH, United States; ^6^ University Hospitals-Cleveland Medical Center, School of Medicine, Case Western Reserve University, Cleveland, OH, United States; ^7^ Louis Stokes Veterans Affairs Medical Center, Cleveland, OH, United States

**Keywords:** O-GlcNAcylation, immune system, inflammation, NF-KappaB, leukemia, immunometabolism, hypertension

Maintenance of immune homeostasis is an intricate biological process involving multiple pathways and molecular mechanisms. One such mechanism is the reversible intracellular post-translational modification, O-GlcNAcylation. It plays a key role in regulating cell signaling, transcription, and translation, nutrient sensing, metabolism, development, normal physiology, and pathology. Altered O-GlcNAcylation of cellular proteins has been implicated in immune dysfunction leading to the development of autoimmune, inflammatory and allergic diseases as well as malignancies of both immune and non-immune cells. How O-GlcNAcylation regulates the immune system in health and diseases is an emerging area of research and the knowledge on the precise role(s) of O-GlcNAcylated proteins in the immune cells and immune responses is limited (1). This Research Topic includes seven original research articles and six comprehensive review articles covering a broad area on the role of O-GlcNAcylation in the immune system.

As an apt prelude to this Research Topic, Mannino et al., has compiled an excellent beginner’s review guide on O-GlcNAcylation as a nutrient sensitive pathway with significant impact on the immune system. This review provides sufficient details on the enzymes regulating O-GlcNAcylation, O-GlcNAc transferase (OGT) and O-GlcNAcase (OGA), and present O-GlcNAcylation as a nutrient sensing rheostat in the cell. It also discusses how O-GlcNAcylation regulates protein function directly as well as through crosstalk with other protein modifications with implications in the immune system’s function, autoimmune and inflammatory diseases as well as immune cell malignancy.

Further elaboration on the role of O-GlcNAcylation in immune cell malignancy is provided in the review by Spaner and the original articles by Shu et al., and Schauner et al. Spaner has comprehensively reviewed the effects of O-GlcNAcylation on oncogenic signaling pathways in Chronic Lymphocytic Leukemia (CLL), calling out key regulators such as p53, AKT, NF-kB, RAS, WNT, NOTCH, MYC and STAT proteins, as well as CLL metabolism. Roles of T cells and tumor associated macrophages in CLL are also discussed including details on associated mechanisms. How aberrant O-GlcNAcylation affects other hematological malignancies such as acute myeloid leukemia (AML), acute lymphoblastic leukemia (ALL), mantle cell lymphoma (MCL), diffuse large cell lymphoma (DLCL) and multiple myeloma (MM) are also discussed. Informative debates on the functions of O-GlcNAcylation as a tumor promoter or a tumor suppressor and notes on options to block or enhance O-GlcNAcylation as potential therapeutic approaches to treat cancer adds great value to this elegant review.

Shu et al., presents novel findings on the correlation between epidermal growth factor (EGF) domain-specific OGT (EOGT) expression and immune infiltration. Their results show elevated EOGT expression in tumor infiltrating immune cells, which leads to multiple immune defects including exhausted T cells and immune suppressor cells and lower proportion of cytotoxic T cells in hepatocellular carcinoma (HCC). A large set of bioinformatics data is presented showing EOGT expression in HCC along with immunofluorescent staining of EOGT in normal liver tissue and HCC as validation. Relevant details including different subgroups of EOGT, insight into its biological function, protein-protein interaction network and association between EOGT and immune cell markers are also presented. Although EOGT causes an atypical O-GlcNAcylation in endoplasmic reticulum, it also depends on hexosamine biosynthetic pathway (HBP) and UDP-GlcNAc to modify serine and threonine residues of target proteins. Thus, this study suggests that knowledge on EOGT mediated O-GlcNAcylation may prove relevant to gain a holistic view on the role of HBP and O-GlcNAcylation in immune cell function and cancer.

Schauner et al., present cutting edge research on O-GlcNAcylation at single cell level in AML. This study shows that expression of key enzymes regulating O-GlcNAcylation are elevated in AML cells compared to healthy blood cells at single cell level and bulk cells. This is a seminal study in this area where they specifically compared HBP gene expression in AML stem cells and blasts and validated RNA expression data using flow cytometry-based analysis of global O-GlcNAcylation and OGT expression in AML patient cells. Gene set analysis and functional studies identified O-GlcNAcylation-dependent activation of NF-kB pathway as a mediator of cell survival and proliferation in AML. This study also provides key knowledge on the potential of blocking HBP and O-GlcNAcylation in AML as a direct or combination therapy approach to control AML and suggests targeting this pathway in stem cells may control relapse.

The articles by Abramovitz et al., Dong et al., and Feinberg et al., direct the attention to the role of O-GlcNAcylation in innate immunity and inflammation. Abramovitz et al., studied the role of O-GlcNAcylation in macrophage function, a primary innate immune mechanism that sense the local microenvironment and responds to harmful stimuli. They specifically examined how enhanced O-GlcNAcylation affects proinflammatory M1 macrophages and found enhanced expression of cytokines IL-1, IL-6 and IL-12, that may promote inflammation. They also discovered inducible nitric oxide synthase (iNOS) as an OGT target, and the O-GlcNAcylation-dependent interplay between nitric oxide production and inflammation maintaining macrophage homeostasis. Dong et al., reviewed the role of O-GlcNAcylation in innate immune cell functions and elaborated on how it regulates acute inflammation and antiviral immune response. They focused on the regulation of NF-kB signaling pathway by O-GlcNAcylation with details on the specific O-GlcNAcylation site-function relationship on key proteins such as RelA, c-Rel, IKKb, and TAB1, all of which are involved in the regulation of inflammatory response. They also summarized information on how O-GlcNAcylation of other regulators such as STAT3 and RIP3K as well as OGT binding to transcriptional co-repressor complex will affect inflammatory signaling. Brief notes are also provided on the contradictory findings on inflammatory responses observed in OGT and OGA knockout mouse models. Key roles of O-GlcNAcylation in host defense against viruses are concisely presented with reference to both RNA viruses (influenza A virus, Sendai virus, vesicular stomatitis virus and hepatitis C virus) and a DNA virus (Hepatitis B virus), with relevant details on mechanisms involved in antiviral immunity and T cell immune response. Feinberg et al., studied the cell type specific role of O-GlcNAcylation in the cytotoxicity of natural killer (NK) cells. While most studies in the literature describes glucose metabolism and stress dependent O-GlcNAcylation, this study shows that stimulation of NK cells with the cytokines, IL-2 and IL-15 enhances O-GlcNAcylation, which is necessary for the expression of NK cell receptors, inflammatory cytokines and mediators of death in NK cells. Providing physiologically relevant evidence, inhibition of O-GlcNAcylation was shown to prevent the ability of NK cells to kill tumor cells *in vitro* and *in vivo*.

Mukherjee et al., have presented rather less studied, but highly interesting role of O-GlcNAcylation in antigen presentation. They performed a mass spectrometry-based approach using B lymphoblastoid cell line JY to identify HLA Class I peptides modified by O-GlcNAcylation and presented a complied list of modified peptides identified in their study and those described in the literature. This provides valuable information on posttranslationally modified peptides in cancerous and non-cancerous cells revealing several peptidoforms generated through O-GlcNAcylation, phosphorylation and methylation as well as glycan heterogeneity. Discussion on the plausible functional diversity imparted by peptide modifications on antigen presentation, offers an interesting future direction of research.

The review by Dos Passos Junior et al., brings in a very interesting angle of study, where the role of O-GlcNAcylation is linked to hypertension and its effects on the immune system. Hypertension is a multifactorial disease, and several arms of the immune system has been shown to contribute to its progress and associated diseases. Several factors such as immune cell activation, cellular stress and inflammation have been associated with both hypertension and O-GlcNAcylation, suggesting the possibility of targeting O-GlcNAcylation as a potential approach to control hypertension. This in-depth review describes how O-GlcNAcylation affects arterial hypertension, myocardial dysfunction, cardiac hypertrophy and other heart diseases citing multiple experimental models providing a wealth of knowledge. Specific sections discuss the link of O-GlcNAcylation in innate and adaptive immune pathways to hypertension warranting the necessity of future research on this unique topic.

Because O-GlcNAcylation depends on glucose availability, it holds a tight link to cellular metabolism as a whole and several recent studies have contributed to the emergence of a new direction of metabolic research, i.e., immunometabolism (2, 3). Adding another piece of knowledge to the field of immunometabolism, Zhang et al. presented new studies on how O-GlcNAcylation of STAT5 controls energy and iron metabolism in regulatory T cells. Their Treg cell specific OGT knockout mouse model shows that Treg cell O-GlcNAcylation is essential to control adiposity and insulin resistance. They also show that O-GlcNAcylation-dependent STAT5 function in Treg cells regulates iron metabolism and propose that targeting O-GlcNAcylation-STAT5 axis and thereby modulating Treg cell function may prove effective in treating obesity and metabolic disorders.

The two articles by Xi et al., and Saha et al., deserves special mention as the ones discussing tools to study O-GlcNAcylation. Xi et al., presented a novel shark single domain antibody as a tool for intracellular detection and localization of OGT. They successfully generated anti-human OGT antibodies in shark and convincingly demonstrated its use to detect OGT by ELISA, Western blotting, flow cytometry and immunofluorescence. Considering the limited availability of tools to study O-GlcNAcylation in cells, this is an appreciable contribution to the field. Saha et al., summarized the currently available chemical tools to study O-GlcNAcylation in the immune system. They have reviewed the tools used to study O-GlcNAcylation describing the effects of OGA inhibitors- PUGNAc, Thiamet-G and GlcNAcstatin-G and OGT inhibitors- Ac_4-_5S-GlcNAc and OSMI-1, on various signaling pathways controlling immune cell functions. Discussions also include strategies available for metabolic and chemoenzymatic O-GlcNAc labeling using Ac_4_GalNAz and UDP-GalNAz as the nucleotide donor for mass spectrometry studies, as well as IsoTaG labeling involving click-chemistry. They conclude by saying that improved tools are needed to study O-GlcNAcylation *in vivo* and a combination of chemical and genetic approaches to target O-GlcNAcylation is expected to advance immunotherapeutic development.

Emphasizing the substantial clinical relevance of the knowledge on O-GlcNAcylation in the immune system, Cai et al., have reviewed how O-GlcNAcylation regulates function of individual immune cell types and its therapeutic potential. They have discussed the O-GlcNAcylation-dependent molecular mechanisms controlling immune cells highlighting its functions in monocytes, macrophages, dendritic cells, neutrophils, NK cells, T cells and B cells. They have also included discussion on the role of O-GlcNAcylation in cardiovascular diseases and cancer mentioning known proteins and O-GlcNAcylation site specific functions.

Despite covering several aspects of the immune functions, this Research Topic is limited in discussing the roles of O-GlcNAcylation in certain key areas such as immunological memory and autoimmune diseases. Changes in O-GlcNAcylation have been linked to various autoimmune conditions. For instance, altered O-GlcNAcylation patterns have been observed in diseases like rheumatoid arthritis (4) and systemic lupus erythematosus (SLE) (5), potentially contributing to disease pathology by affecting immune cell function and inflammation. Considering its role in B and T cells, it is also conceivable that O-GlcNAcylation will have critical roles in regulating immunological memory. Similarly, since O-GlcNAcylation is responsive to cellular stress and nutrient availability (6, 7), it is also expected to modulate immune responses under stress conditions, such as during infection or inflammation. Based on the current knowledge, it appears that the development and testing of protein-specific O-GlcNAcylation-modulating drugs holds high scope to treat immune disorders. Emerging new diagnostic tools that use O-GlcNAcylation patterns to assess immune system status or disease progression, may enable earlier and more accurate disease diagnoses.

In summary, this collection of thirteen articles together with the cited one thousand and thirty-seven references provides a comprehensive current view of the plethora of functions controlled by O-GlcNAcylation in health and disease in the immune system. It also draws attention toward unexplored areas that warrant future research, poised to offer new insights into immune regulation and potential therapeutic strategies.
